# Occlusion of thoracic duct stent resulting in recurrent chyluria: role of renal-lymphatic fistula embolization

**DOI:** 10.1186/s42155-023-00387-6

**Published:** 2023-08-07

**Authors:** Tran Quoc Hoa, Nguyen Ngoc Cuong, Le Hoan, Nguyen Hoang, Hoang Long, Doan Tien Luu, Nguyen Cong Hoan

**Affiliations:** 1 Surgery Urology, Hanoi Medical University Hospital, Hanoi, Vietnam; 2 Radiology Center, Hanoi Medical University Hospital, Hanoi, Vietnam; 3 Diagnostic Imaging and Interventional Center, Hanoi Medical University Hospital, No1, Ton That Tung, Dong Da, Hanoi, Vietnam; 4 Respiratory Department, Hanoi Medical University, Hanoi, Vietnam; 5 Surgery Department, Hanoi Medical University, Hanoi, Vietnam; 6 Outpatient Department, Hanoi Medical University Hospital, Hanoi, Vietnam

**Keywords:** Chyluria, Lymphangiography, Thoracic duct, Stent, Occlusion, Embolization

## Abstract

**Background:**

Thoracic duct (TD) stenting is considered a treatment option for certain pathological conditions caused by TD obstruction, such as chyluria. Several studies have reported on the efficacy of TD stent treatment for both obstructive and leakage condition of TD, but few have evaluated the stent patency. This report aims to describe the patency of TD stent and the effectiveness of renal-lymphatic fistula embolization in the treatment of chyluria.

**Case presentation:**

We report a case of chyluria treated by TD stent previously, stent was placed at the TD venous junction four months before the symptoms recurred. At the second intervention we found the stent was obstructed by debris. We recanalized the stent and successfully catheterised the microcatheter through the stent retrograde into the TD then into the renal-lymphatic fistula branch. After embolization of that abnormal branch, the recurrent chyluria was treated and no further episode of chyluria was occurred during 12 months follow up.

**Conclusion:**

Stent in the TD may be occluded by debris. Embolization of renal-lymphatic fistula might be the most important treatment for spontaneous chyluria.

## Introduction

Chyluria is a rare condition caused by trauma, surgery, malignant diseases. Recent advances in lymphatic imaging and intervention have suggested another possible cause of chyluria, which is obstruction of the thoracic duct (TD). This may increase pressure within the TD, leading to the development of collateral lymphatic vessel that drain into the kidney [4]. Currently, there are no standard guidelines for the treatment of chyluria. Traditional treatment based on combination of low-fat diet, octreotide infusion with surgical treatment or injection of silver nitrate into the renal pelvis to sclerose the renal-lymphatic fistula [7].

Recent interventional treatment method that combines embolization of renal-lymphatic fistula with TD balloon plasty or stenting have successfully treated, but only few cases have been described [2, 4, 5]. Aside from chyluria, impeded flow in the TD is believed to be a cause of various pathological conditions, such as chylous ascites, chylous thorax, plastic bronchitis. Therefore, TD stent has been described in the treatment of various other pathologies [3, 8, 9]. To date, few studies described the patency of TD stent. Our report serves two purposes: 1) To provide data on TD stent patency, and 2) To report the role of renal-lymphatic fistula obstruction in the treatment of chyluria.

## Case report

A 64-year-old female patient was diagnosis of chyluria for more than 10 years. Episodes of chyluria occurred intermittently; however, recently the symptoms lasted for several months, necessitating interventional treatment.

**Fig. 1:**
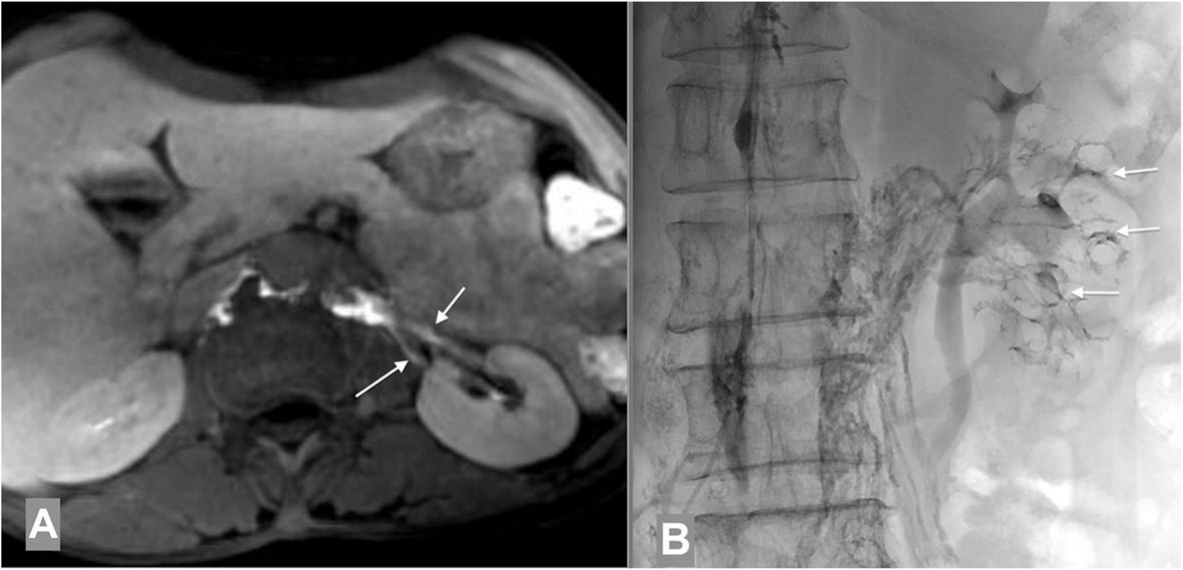
Abnormal lymphatic vessels on the left kidney. **A** Renal – lymphatic fistular on the left kidney seen on MRI (arrows). **B** Multiple tiny lymphatic vessels toward the left kidney and the fistula into the renal collecting system (arrows) seen fluoroscopic image

She underwent MRI lymphangiography which showed the abnormal lymphatic vessels in the left renal pelvis (Fig. 1a). Intranodal lymphangiography was done which showed the stagnation flow of lipiodol (Guerbet, France) in the TD and the presence of abnormal lymphatic vessels toward the left kidney (Fig. 1b).

**Fig. 2:**
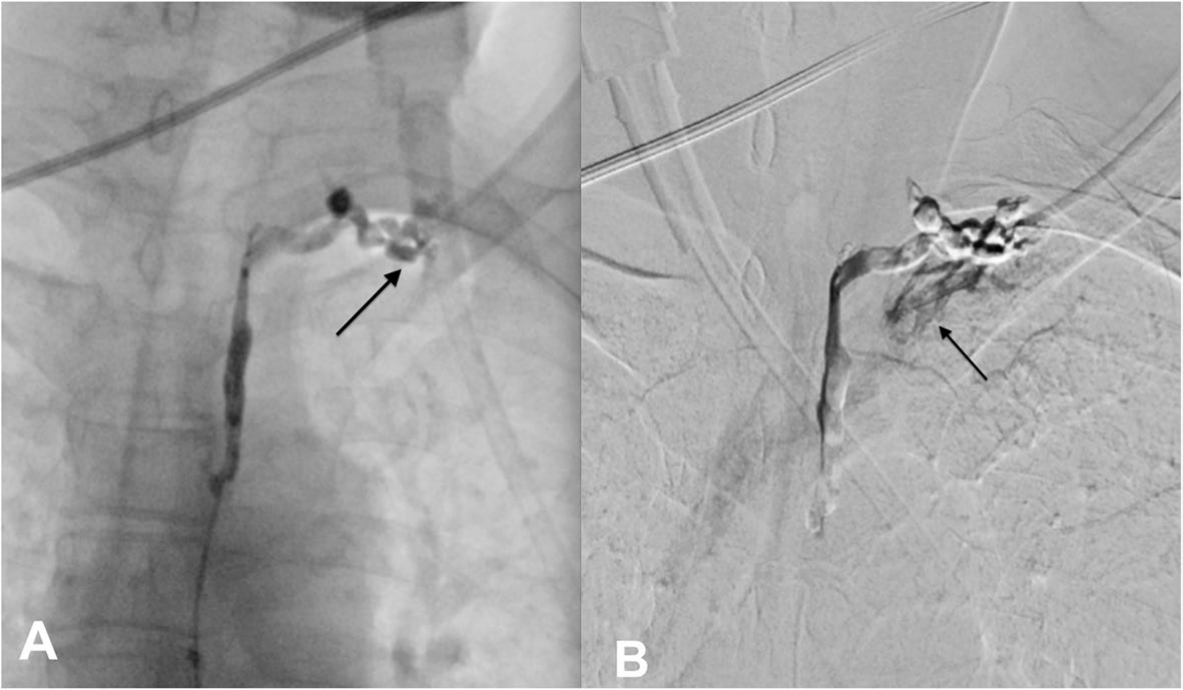
Thoracic ductography through microcatheter placed in the TD. **A** Gentle injection of contrast material showed retention of the contrast at the junction TD subclavian vein (arrow). **B** High pressure injecting contrast showed the flow in the subclavian vein (arrow)

The cysterna chyli was punctured by a 21-gauge needle (Chiba, Cook company, USA) under fluoroscopy. Subsequently, a microcatheter (Progreat 2.7F, Terumo, Japan) was inserted into the TD as described in the literature [2]. When contrast was injected into the TD though microcatheter with very gentle pressure, there was no flow in the TD and the contrast material stopped at the lympho venous junction (Fig. 2a). Only when the contrast was injected with high pressure under subtraction, the flow at the lympho venous junction could be seen (Fig. 2b). The diagnose was made as there might be an elevation of pressure in the TD causing the reflux of chyle into the left renal pelvis. The patient was put an uncovered drug eluting ballooning stent (Resolute Onyx DES coronary stent, Medtronics, USA) at the terminal part of TD which was described in the literature previously [2]. The interstitial lymphatic vessels embolization was attempted to embolize, but the technique was failed so, no embolization was done to occlude the renal- lymphatic fistula in that patient. However, the chyluria was disappeared after intervention.

**Fig. 3:**
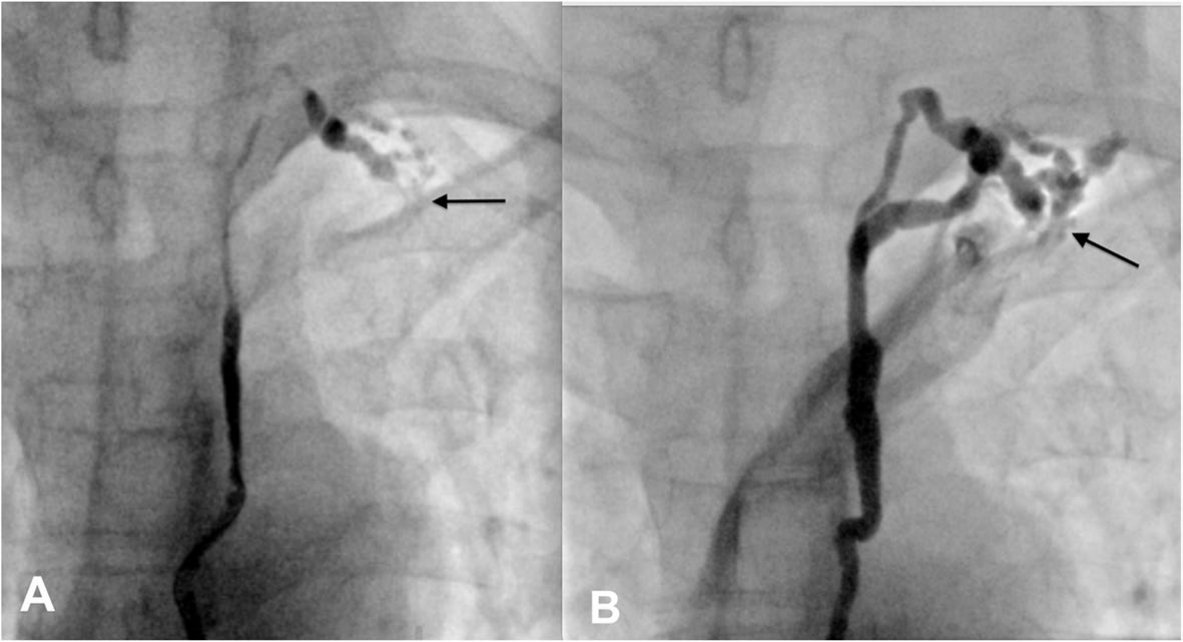
TD stent obstruction. **A** Contrast material stopped at the stent (arrow). **B** Stent flowed after injecting with high pressure (arrow)

**Fig. 4:**
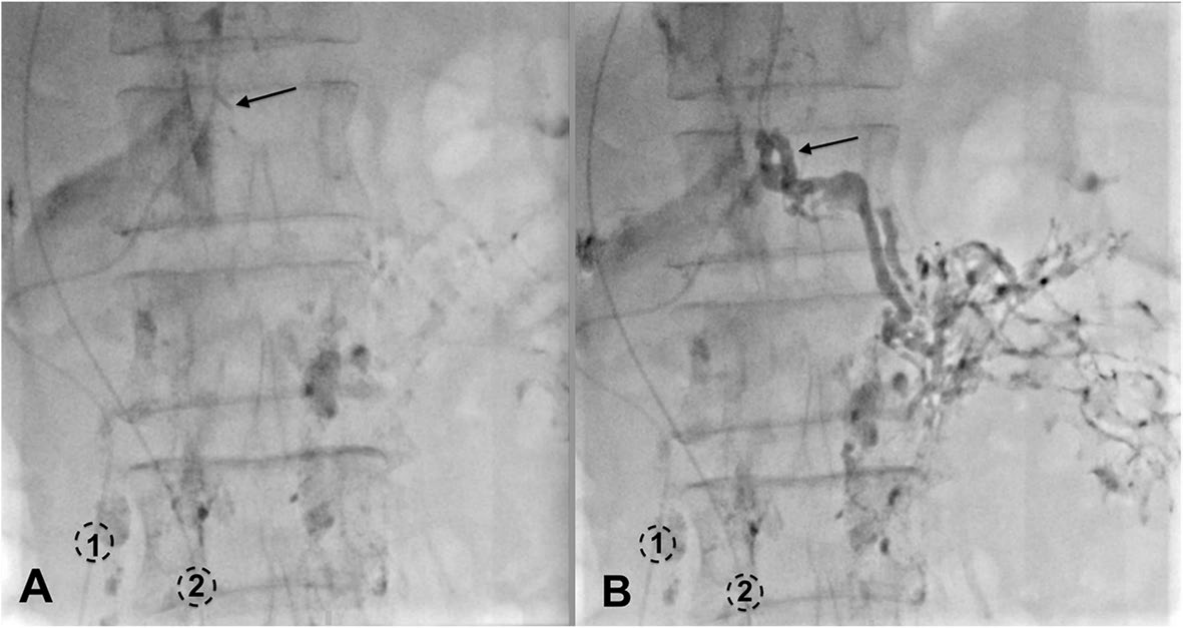
Embolization renal-lymphatic fistular. **A** The first microcatheter (1) was transabdominally placed with its tip was positioned at the lower part of thoracic duct. The 5%-glucose was injected into the first microcatheter while NBCA was being injected from the second microcatheter **B**. The second microcatheter (2) was retrogradely placed in the abnormal lymphatic branch (arrow). During embolization of renal-lymphatic fistula, glue was filled in the abnormal lymphatic branches at the left renal hilar, while the thoracic duct remained patent without glue

Four months later, the patient represented because the symptoms of chyluria recurred. Intranodal lymphangiography was repeated to verify the flow in the thoracic duct stent and tried to embolise the renal-lymphatic fistula. On lymphangiography, slow lymphatic anterograde was seen as the previous intervention. The cisterna chyli was punctured and inserted the microcatheter successfully. When injecting the contrast material through the microcatheter, no flow in the stent at the gentle pressure (Fig. 3a). However, when we increased the injected pressure, the flow passing the stent was seen (Fig. 3b). Because the renal-lymphatic fistular could not be embolized at the previous intervention by direct puncture, we tried to embolize it retrograded access. Through the first microcatheter in the TD, we advanced the 0.014″-guide wire into the thoracic duct (trancend 300, Boston scientific, USA), then passed the stent into the subclavian vein and into the inferior vena cava. Through a snare which was placed at the inferior vena cava, the guide wire was caught out to the right femoral vein. From femoral vein, a second microcatheter (Tokai, Camelian, Japan) was advanced into thoracic duct through the 0.014″-guide wire. When the second microcatheter was at the thoracic duct retrogradely, contrast material was injected to see the abnormal lymphatic branch into the left renal pelvis. Then, the second microcatheter was selectively canulated the abnormal branch while the first microcatheter was placed at the lower part of TD. The mixture glue NBCA with lipiodol (ratio 1:4) was injected into the abnormal branch while the first microcatheter was injected the glucose 5% parallelly to prevent the reflux of glue into TD (Fig. 4). After occlusion of renal-lymphatic fistula, the symptoms of chyluria disappeared, the patient was followed up for 12 months with no recurrent symptoms.

## Discussion

While stenting the occluded thoracic duct was initially treated chyluria, however, the stent was occluded after 4 months resulting in recurrent symptoms. On repeat lymphangiogram, the renal-lymphatic fistula was retrogradely accessed through the recanalized the thoracic duct stent and fistula was emolization with a 1:4 mixture of NBCA glue:lipiodol. No further episodes of chyluria occurred within 12 months follow up.

Thoracic duct obstruction can lead to various clinical consequences with different symptoms, such as abdominal distension, nausea, chylothorax, chylous ascites, bilateral lower leg lymphedema, and chyluria. Kariya et al. reported a case of spontaneous chylothorax and chylous ascites that were resolved after treatment with percutaneous balloon plasty [6]. Gurevich et al. reported a case of chyluria with thoracic duct obstruction caused by compression from a variant anatomy of the brachiocephalic artery [4]. Our patient's symptoms of chyluria completely disappeared after initial treatment with a TD stent, confirming the hypothesis that TD obstruction can cause chyluria.

Recent reports have described the placement of TD stents in individual patients involving TD lesions related to both obstruction and leakage [9]. Chick et al. reported a case of TD stent graft for the treatment of spontaneous chylothorax [1]. After the stent placement, the chylothorax was promptly treated, but the mechanisms for resolving it were not clearly understood. The authors did not ascertain whether the clinical success was due to the obstruction of TD branches or increased flow within the thoracic duct. Cuong et al. reported a case of thoracic duct stent placement for the treatment of chyluria, but the exact mechanisms of clinical success were also unclear, whether it was due to the recanalization of the TD or the embolization of renal lymphatic fistula [2]. In some cases, patients with cirrhosis ascites have also been successfully treated by placing stents to increase circulation in the thoracic duct [3, 8].

The flow within TD stents has been described in a few reports so far. The reason is that to evaluate the flow within the stent, contrast agent needs to be injected into the TD, which involves an invasive procedure. Patients who have undergone successful treatment with TD stent do not require the invasive procedure of puncturing the TD and injecting contrast material. Only patients with recurrent symptoms need to undergo re-intervention. Therefore, through this re-intervention the flow within the stent is assessed, as in the case of our patient and the patients reported by McGregor et al. [8]. In patients who have achieved clinical success after TD stent treatment, it remains unclear whether the stent facilitates the flow of TD or if the occlusion of collateral lymphatic branches occurrs as a result of the stent placement. Our patient is the first reported case of bare stent occlusion.

TD stent graft obstruction has been reported. McGregor et al. reported a case with stent graft obstruction due to thrombus within the venous segment [8]. In this case, the patient underwent treatment with balloon angioplasty and the placement of an extended stent graft connecting the previous stent to the right atrium. Follow-up imaging conducted 3 months after the intervention revealed that the stent remained patent. In our patient, we placed a bare stent, and we believe that stent obstruction was caused by fatty debris. This belief is supported by the fact that the stent easily recanalized when flushed with contrast agent, without requiring balloon angioplasty. Currently, there have been no long-term studies available that describe the patency of TD stents.

Regarding the treatment of chyluria, to date, there is no standardized method. However, clinical case reports have shown that if the embolization is correctly targeted to the renal-lymphatic fistula, this condition can be effectively treated. Gurevich et al. performed anterograde interstitial lymphatic embolization by puncturing the lumbar lymphatic vessels then injecting the glue [4]. Hur et al. used retrograde catheterization from the thoracic duct to embolize the renal-lymphatic fistula [5]. Our patient experienced symptom resolution after TD stent placement, but after 4 months, the stent became obstructed, and the symptoms recurred. Complete resolution of chyluria symptoms was only achieved after embolization of the renal-lymphatic fistula using the retrograde catheterization technique. To acess in the TD retrogradely, the microcatheter may be catheterized through the left upper arm veins (basilic or cephalic), the femoral vein or even through direct puncture, depending on the favourable anatomy or experiences of operators. There is no standard entry point recommended. Regarding the risk of glue leaking into the collecting system, there is no evidence of the occlusion of collecting systems caused by glue. In our patient, the endpoint of injecting the glue was when the reflux of glue into the thoracic duct was seen on fluoroscopy, so there may be small amount of glue leaking into the collecting system. However, no occlusion of collecting system happened.

## Conclusion

TD stent and renal-lymphatic fistula embolization could successfully treat chyluria.

## Data Availability

All data are referenced from the medical records and stored in the hospital.

## Abbreviation

TD: Thoracic duct
